# Non-Invasive Biomarkers for Duchenne Muscular Dystrophy and Carrier Detection

**DOI:** 10.3390/molecules200611154

**Published:** 2015-06-17

**Authors:** Mónica Alejandra Anaya-Segura, Froylan Arturo García-Martínez, Luis Ángel Montes-Almanza, Benjamín-Gómez Díaz, Guillermina Ávila-Ramírez, Ikuri Alvarez-Maya, Ramón Mauricio Coral-Vázquez, Paul Mondragón-Terán, Rosa Elena Escobar-Cedillo, Noemí García-Calderón, Norma Alejandra Vázquez-Cardenas, Silvia García, Luz Berenice López-Hernández

**Affiliations:** 1 Research Center in Technology and Design Assistance of Jalisco State (CIATEJ, AC), National Council of Science and Technology (CONACYT), Guadalajara 44270, Mexico; E-Mails: monica207383614@gmail.com (M.A.A.-S.); ialvarez@ciatej.mx (I.A.-M.); 2 National Medical Centre “20 de Noviembre”, Institute for Social Security of State Workers, Mexico City 03100, Mexico; E-Mails: elfroy77_@hotmail.com (F.A.G.-M.); wicho_unam@hotmail.com (L.A.M.-A.); p.mondragonteran@gmail.com (P.M.-T.); rolasil@yahoo.com.mx (S.G.); 3 National Institute of Rehabilitation, Mexico City 14389, Mexico; E-Mails: bgodiaz@gmail.com (B.G.-D.); rescobarmex@gmail.com (R.E.E.-C.); 4 Faculty of Medicine, National Autonomous University of Mexico, Mexico City 04510, Mexico; E-Mail: guilleavila2000@yahoo.com; 5 Studies Section of Postgraduate and Research, School of Medicine, National Polytechnic Institute, Mexico City 11340, Mexico; E-Mail: rmcoralv@gmail.com; 6 Asociación de Distrofia Muscular de Occidente A.C., Guadalajara 44380, Mexico; E-Mail: dranoemigarcia@gmail.com; 7 Mexican Institute of Social Security-CMNO, Guadalajara 44340, Mexico; 8 Faculty of Medicine, Autonomous University of Guadalajara, Guadalajara 45129, Mexico; E-Mail: avc200180@yahoo.com.mx

**Keywords:** biomarkers, Duchenne, monitoring, MMP-9, MMP-2, TIMP-1, GDF-8, FSTN

## Abstract

Non-invasive biological indicators of the absence/presence or progress of the disease that could be used to support diagnosis and to evaluate the effectiveness of treatment are of utmost importance in Duchenne Muscular Dystrophy (DMD). This neuromuscular disorder affects male children, causing weakness and disability, whereas female relatives are at risk of being carriers of the disease. A biomarker with both high sensitivity and specificity for accurate prediction is preferred. Until now creatine kinase (CK) levels have been used for DMD diagnosis but these fail to assess disease progression. Herein we examined the potential applicability of serum levels of matrix metalloproteinase 9 (MMP-9) and matrix metalloproteinase 2 (MMP-2), tissue inhibitor of metalloproteinases 1 (TIMP-1), myostatin (GDF-8) and follistatin (FSTN) as non-invasive biomarkers to distinguish between DMD steroid naïve patients and healthy controls of similar age and also for carrier detection. Our data suggest that serum levels of MMP-9, GDF-8 and FSTN are useful to discriminate DMD from controls (*p* < 0.05), to correlate with some neuromuscular assessments for DMD, and also to differentiate between Becker muscular dystrophy (BMD) and Limb-girdle muscular dystrophy (LGMD) patients. In DMD individuals under steroid treatment, GDF-8 levels increased as FSTN levels decreased, resembling the proportions of these proteins in healthy controls and also the baseline ratio of patients without steroids. GDF-8 and FSTN serum levels were also useful for carrier detection (*p* < 0.05). Longitudinal studies with larger cohorts are necessary to confirm that these molecules correlate with disease progression. The biomarkers presented herein could potentially outperform CK levels for carrier detection and also harbor potential for monitoring disease progression.

## 1. Introduction

Skeletal muscle tissue provides mechanical strength, confers the ability to move and behaves as a large repository of building blocks for protein synthesis in living beings [1]. DMD is a recessive, chromosome X-linked neuromuscular disorder in which muscle cell integrity is compromised due to the lack of dystrophin, encoded by the *DMD* gene. Clinical features of DMD patients are delayed developmental milestones, frequent falls and progressive muscular weakness; the latter causes disability and subsequent problems such as cardiac [2] and respiratory complications that lead to early death in males. In female carriers only around 8% suffer any manifestations [3], including cardiomyopathy and/or some degree of weakness detected by a cautious clinical examination [4]. Dystrophin is thought to serve as shock absorber molecule, and also as core anchoring element to maintain the cascade flow of extra-cellular signals through an interaction with the Dystrophin Glycoprotein Complex (DGC) in which the dystroglycan complex (DC) (assembled by the α and β dystroglycan proteins) binds dystrophin to form an intracellular link with the cytoskeleton, because dystrophin also has a domain to bind actin [5]. When functional dystrophin is lacking muscles are more sensitive to movement-induced damage, leading to membrane fragility, abnormal calcium influx and activation of proteolytic enzymes; leading to extracellular matrix (ECM) breakdown, necrosis, chronic inflammation and replacement of muscle by endomysial fibrosis and adipose tissue deposits [6]. Several inflammatory molecules and regulators of ECM are disrupted in muscle biopsies from DMD patients and in skeletal muscle of animal DMD models. Among these molecules we can find matrix metalloproteinases (MMPs), which are zinc-containing and calcium-dependent proteases involved in ECM remodeling, inflammation, fibrosis, and activation of various latent cytokines [7,8]. Interestingly, proteins from the DGC are targets for both MMP-9 and MMP-2, therefore are altered in muscular pathologies as well as the natural regulator of MMP-9, TIMP-1 [7]. The inhibition of MMP-9 in *mdx* mice improves proliferation and engraftment of myogenic cells [8]. Both MMP-9 and MMP-2 are able to cleave β-dystoglycan [9], whereas it was shown that MMP-2 exerts proteolytic activity on α-dystroglycan *in vitro* [10]. It was recently suggested that dystrophin is a substrate for MMP-2 in the context of ischemic injury [11].

It was also shown that MMP-9 and TIMP-1 are altered not only in the *mdx* mouse (an animal model for DMD) [12,13,14] but also in serum of DMD patients under steroid treatment [6,15], however serum levels of MMP-2 have not been reported in these patients.

Derived from the loss of dystrophin, other intracellular signaling pathways are also altered such as nuclear factor-kappa B (NF-kB) interacting pathways and the transforming growth factor beta (TGF-β) pathway that negatively affects the regeneration of skeletal muscle through inhibition of satellite cell proliferation, diminished myofiber fusion, and alteration in the expression of some muscle-specific genes. TGF-β1 prompts the transformation of myogenic cells into fibrotic cells after injury [16,17]. A study on DMD, BMD and congenital muscular dystrophy (CMD) showed that plasma levels of TGF-β1 are significantly elevated in DMD and CMD compared to BMD and healthy controls [18]. One of the most notable members of the TGF-β superfamily involved in muscle pathology is GDF-8, that attaches to and activates a complex of activin receptor 2B (Acvr2b) and ALK4 or ALK5 expressed in myogenic stem cells and proliferating myoblasts. Acvr2b receptor activation triggers multiple intracellular signaling cascades including the SMAD and MAPK pathways that stimulate the AKT and p21/Rb pathways and inhibit expression of the muscle regulatory factors (MRFs) [19]. Myostatin can prevent the progress of myoblasts from G1-S phase of cell cycle, maintaining satellite cells quiescent to avoid hypertrophy [19]. Inhibition of GDF-8 by an antibody results in diminished diaphragm pathology in the *mdx* mouse [20]. Interestingly, FSTN, an extracellular antagonist of GDF-8, binds GDF-8 with higher affinity than its receptor Acrv2b, in this manner FSTN could counterbalance muscle loss in DMD.

Currently, no cure for DMD is available, however novel therapeutic strategies to restore dystrophin function are emerging, some with promising results in clinical trials [21,22,23] in which reliable biomarkers and surrogate endpoints of the disease are crucial to evaluate treatment efficacy [24,25].

**Figure 1:**
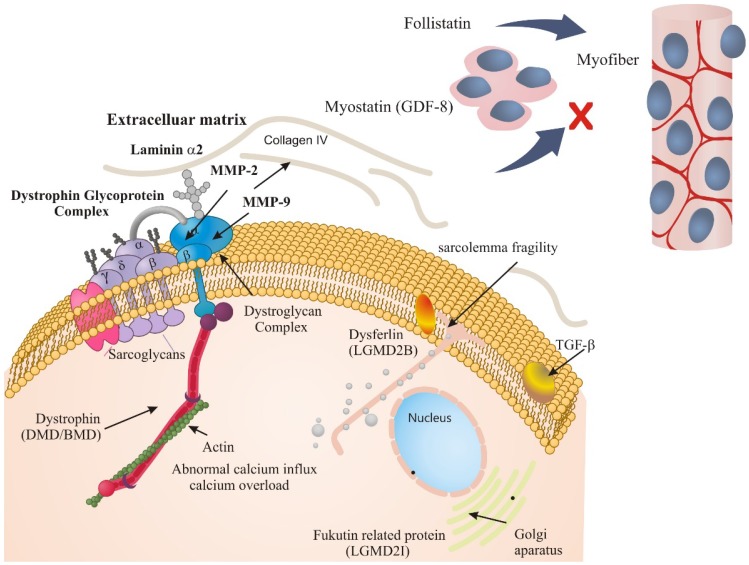
Representation of the dystrophin glycoprotein complex. Dystrophin is depicted in red, while other proteins that cause LGMD2 and LGMD2I such as dysferlin and fukutin-related protein, respectively, are also shown. In the extracellular matrix the cleavage of dytroglycan complex as a target of MMP-2 and MMP9 is shown. As the disease progresses, satellite cell activation occurs to repair damaged muscle, yet nevertheless myofiber differentiation is prevented by GDF-8 and the action of GDF-8 is simultaneously modulated by its inhibitory counterpart FSTN.

Quantification of CK serum level is broadly used as a biomarker for the detection of muscular dystrophies, however, the assay is influenced by age, physical activity and pharmacological treatments among other factors, therefore is not useful for monitoring disease progression or female carrier detection in DMD [26]. Indeed, validated prognostic biomarkers for monitoring disease progression or therapy response are scarce for DMD [6,24,25,27], making necessary biochemical indicators of biological, pathogenic processes or pharmacological responses to therapeutic interventions that could be objectively measured [24]. We hypothesized that GDF-8 that prevents hypertrophy [28,29] and FSTN its inhibitory counterpart [30], together with MMP-2, MMP-9 and TIMP-1 may serve as biomarkers in DMD, so herein we evaluated serum levels of GDF-8, FSTN, MMP-9, MMP-2 and TIMP-1 in DMD steroid naïve patients, patients under steroid treatment and their female relatives in order to assess their potential applicability as non-invasive biomarkers, and trying to refine the role of these biomolecules in the pathology, as it is very crowded and complex (Figure 1). In addition patients with BMD and LGMD muscular dystrophies were included to explore the potential of the abovementioned proteins for differential diagnosis of muscular dystrophies.

## 2. Results and Discussion

### 2.1. ECM Regulators in Duchenne Muscular Dystrophy

Both MMP-2 and MMP-9 are thought to participate in the pathology of dystrophin-deficient skeletal muscle at different stages. MMP-9 may be predominantly involved in the inflammatory course during muscle degeneration, whereas MMP-2 is activated in regenerating fibers associated with ECM remodeling during muscle regeneration and fiber growth [12,13,31]. In addition, TIMP-1 binds with high affinity to the inactive pro-MMP-9, forming a complex. The transcription of TIMP-1 gene is induced by pro-inflammatory cytokines (IL-1, IL-6, OSM, LIF and TNF-α) and TGF-β1 [32,33,34] which are expected to be increased in DMD due to the presence of fibrosis.

**Figure 2:**
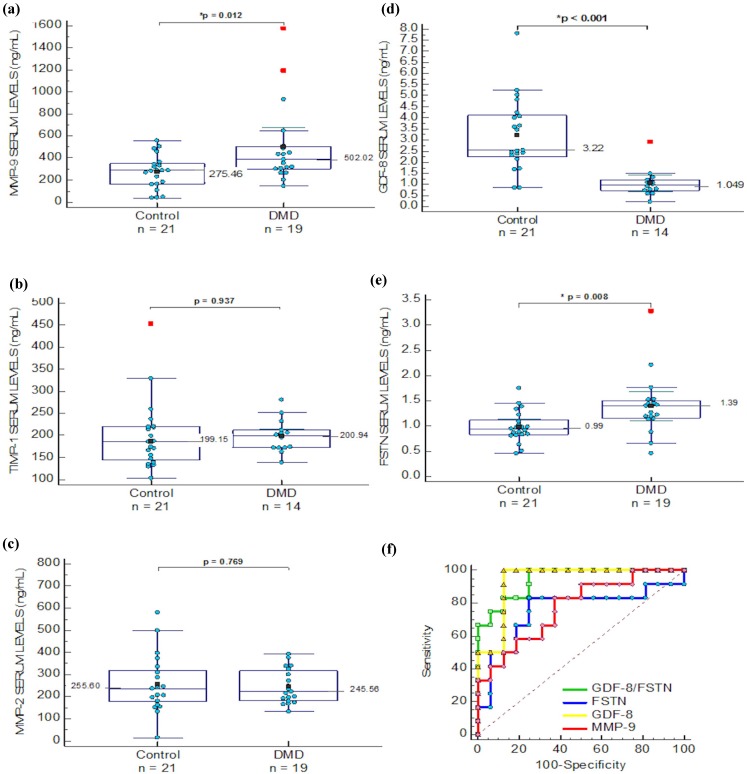
Comparison of serum levels between groups (**a**) MMP-9 levels; (**b**) TIMP-1 levels; (**c**) MMP-2 levels; (**d**) GDF-8 levels; (**e**) FSTN levels; (**f**) Comparison between GDF-8 (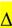), FSTN (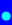), MMP-9 (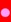) and GDF-8/FSTN ratio (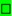) levels ROC analysis.

Unlike the report by Nadarajah and colleagues that included patients under steroid treatment [6], in the present study, serum levels of MMP-9, MMP-2 and TIMP-1 were analyzed in a DMD group of steroid naïve ambulant patients (*n* = 19, age = range 3–12; mean and SD = 8.2 ± 2.1 years) and in a control group of healthy male children (*n* = 21, age = range 5–13; mean and SD 9.9 ± 2.5 years) (Figure 2). When MMP-9 means were compared, there was a 2-fold increase in DMD patients, whereas TIMP-1 and MMP-2 did not show differences (Figure 2a–c and Table 1). In addition, case-by-case correlation analysis of MMP-2, TIMP-1 and MMP-9 was performed with age and clinical variables, including functional tests to evaluate motor ability in DMD. Interestingly, MMP-9 values showed direct correlation with the time to perform the Gowers’ maneuver, indicating that patients with higher levels of MMP-9 perform slower movements (*p* < 0.05). Similarly, in the 10 meters walk test, patients with higher levels of MMP-9 required more time to perform the test (*p* < 0.05). In addition, MMP-2 serum levels correlated inversely with the time to rise from the chair (correlation coefficient = −0.586, *p* = 0.02) (Table 2). Remarkably, the MMP-9/TIMP-1 ratio correlates with Barthel index (R = −0.829, *p* = 0.01), Brooks (inferior) (R = 0.866, *p* = 0.001), time for Gowers’ maneuver (0.97, *p* < 0.001) and time to put a shirt (R = 0.83, *p* = 0.006).

**Table 1:** Comparison of protein serum levels in DMD, BMD and female carriers (matched by age).

Protein	DMD			BMD			Carriers		
	Patients *n* = 19 Mean (±SD)	Controls *n* = 21 Mean (±SD)	*p* Value	Patients *n* = 4 Mean (±SD)	Controls *n* = 4 Mean (±SD)	*p* Value	Carriers *n* = 17 Mean (±SD)/Median (Range)	Controls *n* = 17 Mean (±SD)/Median (Range)	*p* Value
**MMP-9**	502.021 (174.297)	275.46 (68.62)	0.012 *	785.05 (180.9)	165.3 (114.18)	0.001 *	590.48 (134.79)	705.76 (155.62)	0.244
**MMP-2**	245.56 (37.69)	255.60 (58.40)	0.769	254.76 (32.98)	519.83 (112.93)	0.004 *	ND	ND	ND
**FSTN**	1.39 (0.28)	0.99 (0.14)	0.008 *	1.02 (0.152)	0.715 (0.152)	0.028 *	^¶^ 0.992 (0.516–1.259)	^¶^ 1.833 (0.934–2.702)	0.008 *
**GDF-8**	^#^ 1.049 (0.364)	3.22 (0.752)	≤0.01 *	ND	ND	ND	2.095 (0.726–4.354)	2.886 (1.272–3.974)	0.042 *
**TIMP-1**	^#^ 200.94 (22.3)	199.15 (35.5)	0.937	ND	ND	ND	239.5 (142.29–785.1)	220.93 (124.7–448.7)	0.558

Note: * stastistical significance, ^#^ *n* = 14, ^¶^ *n* = 9.

**Table 2:** Correlation between levels of biomarkers and clinical parameters of steroid naïve DMD ambulant patients. ** Correlation is significant at the 0.01 level (two-tailed). * Correlation is significant at the 0.05 level (two-tailed).

Biomarker		Age (Years)	NSAA	6 MW	Barthel	Brook L	Vignos	T10mw	T10mr	Gowers	Stair	Chair	Shirt
**MMP-9**	Correlation coefficient	0.13	−0.34	0.16	0.05	0.29	0.39	0.2	0.650 **	0.514 *	0.21	−0.12	0.39
	*p*-Value	0.58	0.25	0.61	0.85	0.21	0.09	0.56	**0.01**	**0.04**	0.43	0.62	0.11
**TIMP-1**	Correlation coefficient	−0.24	0.68	−0.23	0.07	−0.27	−0.07	0.09	0.05	0.09	0.39	−0.37	−0.09
	*p*-Value	0.44	0.06	0.62	0.86	0.38	0.82	0.82	0.89	0.81	0.29	0.32	0.81
**GDF-8**	Correlation coefficient	0.02	−0.13	-0.3	−0.33	−0.22	−0.16	−0.27	−0.36	−0.13	−0.09	0.29	−0.07
	*p*-Value	0.95	0.75	0.4	0.32	0.47	0.61	0.42	0.25	0.7	0.8	0.39	0.84
**MMP-2**	Correlation coefficient	−0.1	0.41	0.13	0.3	−0.3	−0.37	0.29	0.03	−0.25	−0.17	−0.586 *	−0.41
	*p*-Value	0.68	0.16	0.67	0.37	0.22	0.13	0.41	0.9	0.39	0.55	**0.02**	0.11
**FSTN**	Correlation coefficient	−0.22	−0.14	−0.02	0.02	0.30	0.03	0.19	0.17	0.00	0.1	−0.07	0.16
	*p*-Value	0.38	0.64	0.94	0.94	0.21	0.91	0.59	0.51	0.99	0.73	0.79	0.56

### 2.2. Matrix Metalloproteinases in Other Muscular Dystrophies

In order to test if MMP-9 and MMP-2 were different in other muscular dystrophies, a group of BMD patients was included as well as a group of LGMD. The comparison of BMD (*n* = 4) *vs.* age matched healthy controls (*n* = 4) showed that MMP-9 was increased in patients (*p* = 0.001). Likewise, healthy controls had higher serum levels of MMP-2 than BMD patients (*p* = 0.004). In the LGMD group MMP-9 showed no differences between patients (*n* = 3) and healthy controls of similar age (*n* = 4) (*p* = 0.404); whereas MMP-2 was shown to be higher in serum of healthy controls *versus* patients (*p* = 0.013) (data shown in Supplemental Material).

### 2.3. Muscle Growth Regulators in Dystrophinopathies and Other Muscular Dystrophies

Since cycles of muscle damage and regeneration are common in DMD, we hypothesized that serum levels of GDF-8 and FSTN, known as key regulators of muscle growth, would be altered in patients and female carriers of the disease. When healthy controls were compared to DMD ambulant steroid naïve patients, FSTN and GDF-8 were significantly increased and decreased, respectively, in the DMD group (Figure 2d,e). The GDF-8/FSTN ratio was also different between these groups (*p* < 0.05). Then we analyzed a group of patients that underwent deflazacort treatment altogether with the DMD steroid naïve group and healthy controls using ANOVA. FSTN serum levels decreased (*p* = 0.05) as GDF-8 levels increased (*p* = 0.005) in the deflazacort group. The GDF-8/FSTN ratio in the steroid naïve group was similar to the deflazacort group (data not shown). GDF-8 also correlated with age, only in the healthy control group (*p* < 0.05). No other correlations were found for GDF-8 and FSTN in the DMD steroid naïve group, including functional assessments (Table 2). In addition, for FSTN a comparison of BMD patients (*n* = 4) *vs.* age matched controls, which showed that FSTN was significantly higher in BMD patients (*p* = 0.028); similarly, in LGMD males (*n* = 3) FSTN serum levels were higher in the group of patients compared to healthy controls (*n* = 4), (*p* = 0.016) (data shown in supplemental material).

### 2.4. Serum Biomarkers for Carrier Detection in Duchenne Muscular Dystrophy

In order to find useful diagnostic biomarkers for carrier detection in Duchenne Muscular Dystrophy, we included a female group that comprised confirmed carriers and healthy females. We compared serum levels of MMP-9, TIMP-1 that were previously proposed as biomarkers of DMD [16], GDF-8 and FSTN. MMP-2 was not measured as it did not show differences among patients that have hemizygous phenotype and we did not expect to find differences in the heterozygous state of female carriers. Differences between carriers and healthy females were found only for GDF-8 and FSTN, the last protein being analyzed only in a subset of women in which clinical history could rule out endometriosis and breast and ovary cancer (therefore this group is smaller, *n* = 9). In the healthy control group, MMP-9 correlated directly to BMI (R = 0.489, *p* = 0.046) whereas FSTN correlated inversely with this parameter (R = −0.896, *p* = 0.003) as well as the GDF-8/FSTN ratio that correlated directly to BMI (0.816, *p* = 0.013) but was unable to distinguish between carriers and healthy females (*p* > 0.05). In addition, the GDF-8/FSTN ratio correlated with age (R = 0.729, *p* = 0.026) in the healthy female group. No correlations were found in the carriers group.

### 2.5. Sensibility and Specificity of Serum Biomarkers in DMD Patients and Carriers

For the DMD steroid naïve patient group, with the aim to determine the diagnostic power of each protein that showed significant difference in this study, receiver operating characteristics (ROC) analysis was performed by plotting the rate of true positives (sensitivity) *vs.* false positive (100-specificity) (Figure 2f). These analyses are based on multiple iterations in order to find the best fitting function of probability for a set of possible cutoff points obtained with the samples included in the study.

For MMP-9 levels the ROC curve analysis showed an area under the curve (AUC) of 0.719 (*p* = 0.007), where the association criterion to the DMD group was >289.8 ng/mL, a value that represents the best fitting point for sensitivity and specificity, corresponding to a sensitivity of 78.95 and 61.9 for specificity. For GDF-8 the AUC was 0.915 (*p* < 0.001), with an association criterion for the DMD group ≤1.485 ng/mL; sensitivity and specificity were 92.86 and 90.48, respectively. For FSTN, the AUC was 0.772 (*p* = 0.0008), with an association criterion for the DMD > 1.0922 ng/mL, sensitivity and specificity were 84.21 and 76.19, respectively.

**Figure 3:**
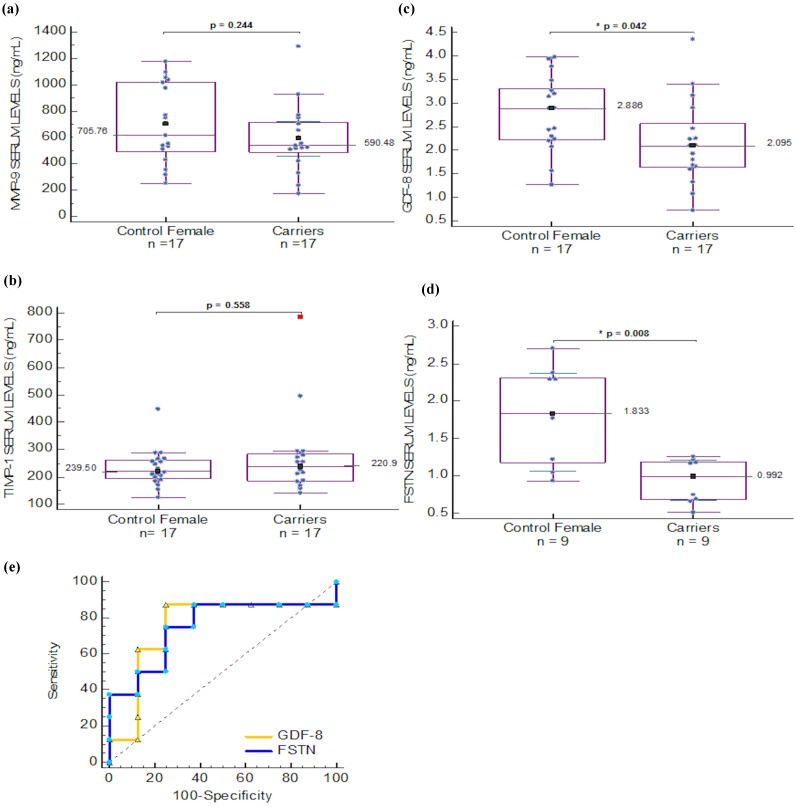
Comparison of (**a**) MMP-9; (**b**) TIMP-1; (**c**) GDF-8 and (**d**) FSTN serum levels in female group; (**e**) ROC curve analysis of GDF-8 (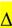) and FSTN (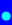) levels in female serum samples.

A non-parametric paired comparison among ROC curves for MMP-9, GDF-8, FSTN, and the ratio of GDF-8/FSTN was performed. There was no difference in the comparison of these AUCs. MMP-9 *vs.* GDF-8 (*p* = 0.084), *vs.* FSTN (*p* = 0.936), *vs.* GDF-8/FSTN ratio (*p* = 0.095). GDF-8 *vs.* FSTN (*p* = 0.113), *vs.* GDF-8/FSTN ratio (*p* = 0.275). FSTN *vs.* GDF-8/FSTN ratio (*p* = 0.054) (Figure 2f). For the female group the ROC curve analysis was also performed for GDF-8 and FSTN (Figure 3e). Where the AUC for GDF-8 was 0.706, *p* = 0.028, with an association criterion for being a carrier as ≤2.2477 ng/mL; sensitivity and specificity were 70.59 and 70.59, respectively. The AUC of FSTN was 0.877, *p* < 0.0001 with an association criterion for the carrier groups was ≤1.217 ng/mL; and sensitivity and specificity of 88.89 and 77.78, respectively. When the comparison between both AUC values was performed, no difference was found (*p* = 0.547).

### 2.6. Discussion

Indeed, CK levels are useful for diagnosis of DMD patients, as it is widely known that specificity of CPK is approximately 94.1% with a sensitivity of 100% in DMD [33], however CK levels are also increased in other muscular dystrophies. Another pitfall in the use of CK levels as biomarker in DMD is that in female carriers, they showed a sensitivity of 33.3% and a diagnostic specificity of 50% [26], hence CK levels are not that useful for carrier detection. Prevention aims to improve carrier detection, genetic counseling and prenatal diagnosis in DMD, therefore it would be of benefit to have reliable diagnostic biomarkers of the disease for males and females and prognostic biomarkers for DMD patients [27,35,36,37].

Exploration of non-invasive biomarkers that are useful for diagnosis, prognosis and/or monitoring response to treatments is of utmost importance for research and clinical practice, in which slight changes in molecules involved in DMD progression, could reflect the effect of novel compounds and also could help in medical decisions regarding steroid treatment. Alterations in serum levels of molecules such as MMP-2, MMP-9 and TIMP-1 are not specific to DMD, and many other diseases present abnormal levels of these proteins, therefore its potential for diagnostic biomarker for differential diagnosis is limited. A study of inflammatory neuromuscular disorders such as polymyositis, inclusion body myositis, chronic inflammatory demyelinating polyneuropathy and multifocal motor neuropathy showed that serum levels of MMP-9 and MMP-2 were elevated and diminished, respectively for all disorders, studied, with exception of polymyositis, whereas TIMP-1 levels were unchanged. It should be noted that after treatment of this inflammatory pathology, the MMP/TIMP serum levels changed, reflecting in this way clinical improvement and relapse [7]. In the particular case of muscular dystrophies, a study showed that TIMP-1 plasma levels were elevated in congenital muscular dystrophy (CMD) as well as in DMD, and these levels were not different between BMD and healthy controls [38]. Nevertheless, a recent study showed that proteins present in plasma and serum may differ on their ability to distinguish among groups of patients according to the analyzed sample [25]. On the other hand, Nadarajah *et al.* reported higher TIMP-1 and MMP-9 levels in the DMD group compared to controls, nevertheless all patients were under steroid treatment [6]. In our study, no differences between MMP-2 and TIMP-1 levels were found in healthy children compared to DMD ambulant steroid-naïve patients. Interestingly, we found that MMP-9 serum levels in DMD patients without steroids are higher than healthy controls of similar age, which resembles the findings of Nadarajah *et al.* in patients under steroid treatment. The abovementioned study also reported correlation to age and time on steroid treatment, although no correlation was found with the NSAA. In spite of this, they postulated MMP-9 as a biomarker of disease progression. Our data certainly show a direct correlation between MMP-9 serum levels and time to perform Gowers’ maneuver and also a direct correlation in the timed 10 m walk test, which further suggests that MMP-9 correlates with disease progression in DMD steroid naïve patients. Interestingly, fibronectin acts as one of the substrates of MMP-9 and has also been proposed as biomarker for DMD [35]. In addition, we found an inverse correlation between MMP-2 levels and time to rise from chair; this finding suggests that serum levels of MMP-2 could also contribute to disease progression in DMD ambulant patients. The ratio MMP-9/TIMP-1 was also useful; it correlated with Barthel index, which has been reported to draw a parallel with the degree of respiratory involvement in Duchenne muscular dystrophy [39].

On the other hand, both FSTN up-regulation and GDF-8 blockade have been proposed as potential therapies for DMD [40] since both participate in mechanisms of muscle wasting in DMD [41]. In a previous independent study GDF-8 serum levels were measured in a cohort of DMD patients, they examined the hypothesis that GDF-8 could be increased in DMD patients’ serum thereby enabling treatment by myostatin blockade. They reported higher GDF-8 levels than the reference values, a control group was not included and the assay recognized the pro-domain of GDF-8, the inactive form of myostatin. They did not find a correlation between GDF-8 and age in DMD patients [42], but when we analyzed their data, indirect correlation with age resulted significant (*p* < 0.05). In our study, we found correlation of GDF-8 and age only in male controls. Another report suggests that GDF-8 is a biomarker for Pompe disease; they observed that the levels of GDF-8 (the inactive form) in serum of Pompe patients were lower than in the control group and FSTN levels were also low but none reached statistical significance [43]. In that study serum levels of GDF-8 increased after enzyme replacement therapy (ERT), probably reflecting muscle regeneration after ERT. Low GDF-8 serum levels were observed in DMD patients as well as in female carriers, therefore GDF-8 would be a versatile biomarker in DMD. To our knowledge no other study has measured the active form of GDF-8 in human serum samples. On the other hand, the increase in FSTN levels observed in our group of DMD patients may partially explain the low levels of GDF-8, since FSTN is able to inhibit GDF-8 by competition [44] and according to data from Awano *et al.*, the inactive form of GDF-8 can also inhibit its active form, these two convergent factors may be involved in the low serum levels of GDF-8 found in DMD patients. It should be noticed that our results correspond to the basal levels of all the proteins studied, but fluctuations in protein serum levels under steroid treatment and correlation to functional scales deserve further research. In the group of patients treated with deflazacort, GDF-8 increased and FSTN levels decreased, so we speculate that the balance between these proteins may play crucial role in the dystrophic phenotype. It should be noted that GDF-8 harbors a glucocorticoid receptor element in its promoter that has been shown to be functional when dexamethasone was administered *in vitro* [45] and *in vivo* [46]. The FSTN serum levels assayed in this study originate mainly in the liver; it has been reported that FSTN is an exercise-induced hepatokine, probably implicated in a muscle-liver cross talk during exercise [44]. Some authors have compared DMD with exercise since both processes require muscle repair, nevertheless in DMD repair process is impaired due to the lack of dystrophin, and therefore the ratio of molecules such as GDF-8 and FSTN could be involved in DMD pathology at least in some stages. In an independent study, increased FSTN levels occurred together with increased inflammation, reduced muscle strength, and low bone mineral density in patients with Chronic Kidney Disease [47], this is in agreement with the results obtained in the present study in which DMD patients have reduced muscle strength and inflammation compared to controls that showed higher levels of serum FSTN. Interestingly the ratio GDF-8/FSTN in steroid-treated patients in our study, was similar to steroid naïve patients that are younger and with better motor functions, therefore we speculate that the homeostasis among atrophy and hypertrophy processes and steroid use in DMD is related to GDF-8/FSTN balance. A diagram showing the release and action of these biomolecules as part of the dystrophin glycoprotein complex is shown in Figure 1.

## 3. Experimental Section

### 3.1. Study Participants

Patients and female relatives attending to the Asociación de Distrofia Muscular de Occidente A.C. (Guadalajara, Jalisco) or Sociedad Mexicana para la Distrofia Muscular A.C. (Mexico City, Mexico) were included in the study according to the institution’s ethical considerations. Biological samples were obtained according to the organization’s ethical guidelines. Participants were referred to our laboratory for DNA testing to confirm diagnosis after clinical evaluation performed by a geneticist. This study was approved by the local ethics committee.

### 3.2. Muscular Dystrophy Patients and Healthy Controls

Twenty five male ambulant patients with definite diagnosis of DMD according to MD STARnet criteria [48] (mutation of each male and other details are shown in the Supplemental Material) were recruited for a steroid management program. According to the complete data and sample availability, subgroups of patients were analyzed for each protein (for the patient group *n* = 25, mean age was 8.2 ± 2.06 years, range 3–12 years). At the time of sampling, patients were without corticosteroid treatment, fasting and were evaluated by rehabilitation specialists after sample collection. After that, they were appointed for follow-up of steroid treatment by a multidisciplinary group from both institutions [49]. As control group thirty-eight male children without significant medical disorders were recruited from healthy-child health care visits, (mean age was 11.3 ± 3.11 years, range 5–17 years). An additional group who underwent steroid treatment was included (*n* = 5, mean age was 10.2 ± 2.16, range 7–13 years). In addition, Becker Muscular Dystrophy patients (*n* = 4) and a group of LGMD (*n* = 7, three males and four females) were also involved for comparisons to test the biomarkers’ capacity to detect different muscular dystrophies; in this groups, five patients were LGMD2B due to dysferlin deficiency and two were LGMD2I (Duchenne-like phenotype with mutations in the *FKRP* gene); age and sex matched controls were included.

### 3.3. Neuromuscular Assessments for Patients with Duchenne Muscular Dystrophy

Before entering the steroid program, patients were evaluated by a rehabilitation specialist. Strength evaluation was performed by timed function tests such as timed 10 m walk, timed Gowers’ manoeuver, time to climb four stairs, time to rise from chair; 6-min walk test and time to put on a shirt were also performed. For the assessment of motor function in specific domains, Vignos lower extremity scale, Brooke upper extremity scale and Barthel Index scores were used. For monitoring of disease progression and response to therapy North Star Ambulatory Assessment (NSAA) was executed [50]. Biological sample collection was done the same day before neuromuscular assessment.

### 3.4. Carrier Detection in DMD Families

In addition, women were recruited; 17 female were carriers and we compared them with 17 female without neuromuscular disorders. All participants were asked to refrain from strenuous exercise 24 h before sample collection. Care was taken in the correct classification of carriers; germ line mosaicism cases were excluded, since in these cases mothers are not expected to present systemic muscle involvement (detectable in serum levels of proteins) but rather a subset of mutated germ cells.

### 3.5. DNA Analysis

Peripheral blood was collected by venous puncture. Genomic DNA was extracted from lymphocytes using the CTAB-DTAB method that uses cationic detergents and ethanol precipitation to avoid salt contamination in the DNA samples [51]. A Nanodrop ND-1000 spectrophotometer (Thermo Fisher Scientific, Wilmington, DE, USA) was used to measure sample concentration; 100 ng and 10 ng of DNA were used to perform MLPA and STR segregation assays respectively.

### 3.6. Multiplex Ligation Dependent Probe Amplification

Genetic screening for copy number variations of all exons of the *DMD* gene was done using Multiplex ligation-dependent probe amplification (MLPA) according to manufacturer’s instructions (P034/P035, MRC-Holland) and analyzed using Genemarker^®^ software, version 1.91 as described before [52].

### 3.7. STR Segregation Analysis

In cases of unknown mutations in DMD patients, dystrophin alteration was observed by muscle biopsy and immunofluorescence. After that, a segregation analysis using intragenic short tandem repeats (STRs) flanking the *DMD* gene was performed for female relatives to determine carrier status.

### 3.8. Immunodetection Analysis

All patients with no mutation detected underwent a muscle biopsy procedure. Skeletal muscle biopsies from patients and control quadriceps were conserved frozen in liquid nitrogen-isopentane. Muscle cryosections were prepared for immunofluorescence for the three dystrophin domains and additional proteins as described previously [53].

### 3.9. Serum Samples

Three mL of serum samples were obtained from venous blood derived from participants using serum separator tubes (BD Vacutainer^®^ catalogue number: 368159); samples were allowed to cloth for 15 min, centrifuged at 3000 rpm for 15 min and conserved at −80 °C until analysis. Care was taken to avoid hemolysis.

### 3.10. Determination of Proteins in Human Serum Samples

Samples were thawed at room temperature; serum levels of the proteins of interest were determined using specific commercial immunoassay kits for each one of them. Human MMP-9 (catalog number DMP900), Human TIMP-1 immunoassay kit (catalog number DTM100), Human MMP-2 Immunoassay kit (catalog number MMP-200), GDF-8/Myostatin Immunoassay kit (catalog number DGDF80, active myostatin form) and the Human Follistatin Immunoassay kit (Catalog Number DFN00); all kits were purchased from R & D Systems (Abingdon, UK). Every assay was carried out in duplicate following manufacturer’s instructions.

### 3.11. Statistical Analysis

Statistical analyses were performed using STATGRAPHICS^®^ Centurion XVI, 16.1.11 version. In order to search differences between groups, data sets were subjected to normality testing using the Shapiro-Wilk method; depending on the normality of data we used the Student’s *t*-test, or the non-parametric alternative Mann-Whitney U test. In the same way parametric correlations were evaluated with the Pearson correlation coefficient and Spearman correlation for non-parametric data. For protein levels with significant differences between groups, ROC curves were generated by plotting sensitivity *vs.* 100-specificity and the area under the curve [54] was calculated with 95% confidence interval (CI), using MedCalc^®^ software, version 14.8.1.

## 4. Conclusions

The search for useful biomarkers in DMD is growing nowadays [25], however, biomarkers with useful diagnostic and prognostic values that also allow therapeutic monitoring are not common. In the present study, we found that serum levels of MMP-2 and TIMP-1 are not capable of distinguishing between healthy controls and DMD ambulant steroid naïve patients, whereas our data support that MMP-9 is a reliable marker for DMD in steroid naïve patients, but not for carrier detection. We showed that MMP-9 correlates with physical condition of DMD steroid naïve patients and the MMP9/TIMP-1 ratio correlates with the Barthel index. Further longitudinal studies could disclose the potential of these biomarkers for monitoring disease progression and response to treatment. Interestingly, patients that underwent steroid treatment had higher levels of GDF-8 and lower levels of FSTN, which is opposite to what is seen in steroid naïve patients. To our knowledge this is the first study measuring the active form of GDF-8, serum levels of MMP-2 and FSTN in DMD steroid naïve patients and correlated to assessments used in routine procedures. Importantly, we propose two novel potential biomarkers with diagnostic value for DMD for carrier detection; FSTN and GDF-8.

## Supplementary Materials

Supplementary materials can be accessed at: http://www.mdpi.com/1420-3049/20/06/11154/s1.

## References

[1] Bhattacharya S., Das A., Dasgupta R., Bagchi A. (2014). Analyses of the presence of mutations in dystrophin protein to predict their relative influences in the onset of duchenne muscular dystrophy. Cell. Signal..

[2] Van Westering T.L., Betts C.A., Wood M.J. (2015). Current understanding of molecular pathology and treatment of cardiomyopathy in duchenne muscular dystrophy. Molecules.

[3] Piko H., Vancso V., Nagy B., Ban Z., Herczegfalvi A., Karcagi V. (2009). Dystrophin gene analysis in hungarian duchenne/becker muscular dystrophy families—Detection of carrier status in symptomatic and asymptomatic female relatives. Neuromuscul. Disord..

[4] Mercier S., Toutain A., Toussaint A., Raynaud M., de Barace C., Marcorelles P., Pasquier L., Blayau M., Espil C., Parent P. (2013). Genetic and clinical specificity of 26 symptomatic carriers for dystrophinopathies at pediatric age. Eur. J. Hum. Genet..

[5] Ervasti J.M., Sonnemann K.J. (2008). Biology of the striated muscle dystrophin-glycoprotein complex. Int. Rev. Cytol..

[6] Nadarajah V.D., Van Putten M., Chaouch A., Garrood P., Straub V., Lochmuller H., Ginjaar H.B., Aartsma-Rus A.M., van Ommen G.J., den Dunnen J.T. (2011). Serum matrix metalloproteinase-9 (mmp-9) as a biomarker for monitoring disease progression in duchenne muscular dystrophy (dmd). Neuromuscul. Disord..

[7] Hurnaus S., Mueller-Felber W., Pongratz D., Schoser B.G. (2006). Serum levels of matrix metalloproteinases-2 and -9 and their tissue inhibitors in inflammatory neuromuscular disorders. Eur. Neurol..

[8] Hindi S.M., Shin J., Ogura Y., Li H., Kumar A. (2013). Matrix metalloproteinase-9 inhibition improves proliferation and engraftment of myogenic cells in dystrophic muscle of mdx mice. PLoS ONE.

[9] Bozzi M., Sciandra F., Brancaccio A. (2015). Role of gelatinases in pathological and physiological processes involving the dystrophin-glycoprotein complex. J. Int. Soc. Matrix Biol..

[10] Sbardella D., Sciandra F., Gioia M., Marini S., Gori A., Giardina B., Tarantino U., Coletta M., Brancaccio A., Bozzi M. (2015). Alpha-dystroglycan is a potential target of matrix metalloproteinase mmp-2. J. Int. Soc. Matrix Biol..

[11] Buchholz B., Perez V., Siachoque N., Miksztowicz V., Berg G., Rodriguez M., Donato M., Gelpi R.J. (2014). Dystrophin proteolysis: A potential target for mmp-2 and its prevention by ischemic preconditioning. Am. J. Physiol. Heart Circ. Physiol..

[12] Kherif S., Lafuma C., Dehaupas M., Lachkar S., Fournier J.G., Verdiere-Sahuque M., Fardeau M., Alameddine H.S. (1999). Expression of matrix metalloproteinases 2 and 9 in regenerating skeletal muscle: A study in experimentally injured and mdx muscles. Dev. Biol..

[13] Miyazaki D., Nakamura A., Fukushima K., Yoshida K., Takeda S., Ikeda S. (2011). Matrix metalloproteinase-2 ablation in dystrophin-deficient mdx muscles reduces angiogenesis resulting in impaired growth of regenerated muscle fibers. Hum. Mol. Genet..

[14] Shin J., Tajrishi M.M., Ogura Y., Kumar A. (2013). Wasting mechanisms in muscular dystrophy. Int. J. Biochem. Cell Biol..

[15] Zocevic A., Rouillon J., Wong B., Servais L., Voit T., Svinartchouk F. (2015). Evaluation of the serum matrix metalloproteinase-9 as a biomarker for monitoring disease progression in duchenne muscular dystrophy. Neuromuscul. Disord..

[16] Allen R.E., Boxhorn L.K. (1987). Inhibition of skeletal muscle satellite cell differentiation by transforming growth factor-beta. J. Cell. Physiol..

[17] Li Y., Foster W., Deasy B.M., Chan Y., Prisk V., Tang Y., Cummins J., Huard J. (2004). Transforming growth factor-beta1 induces the differentiation of myogenic cells into fibrotic cells in injured skeletal muscle: A key event in muscle fibrogenesis. Am. J. Pathol..

[18] Ishitobi M., Haginoya K., Zhao Y., Ohnuma A., Minato J., Yanagisawa T., Tanabu M., Kikuchi M., Iinuma K. (2000). Elevated plasma levels of transforming growth factor beta1 in patients with muscular dystrophy. Neuroreport.

[19] Bradley L., Yaworsky P.J., Walsh F.S. (2008). Myostatin as a therapeutic target for musculoskeletal disease. Cell. Mol. Life Sci..

[20] Murphy K.T., Ryall J.G., Snell S.M., Nair L., Koopman R., Krasney P.A., Ibebunjo C., Holden K.S., Loria P.M., Salatto C.T. (2010). Antibody-directed myostatin inhibition improves diaphragm pathology in young but not adult dystrophic mdx mice. Am. J. Pathol..

[21] Ryan N.J. (2014). Ataluren: First global approval. Drugs.

[22] Voit T., Topaloglu H., Straub V., Muntoni F., Deconinck N., Campion G., De Kimpe S.J., Eagle M., Guglieri M., Hood S. (2014). Safety and efficacy of drisapersen for the treatment of duchenne muscular dystrophy (demand ii): An exploratory, randomised, placebo-controlled phase 2 study. Lancet Neurol..

[23] Bushby K., Finkel R., Wong B., Barohn R., Campbell C., Comi G.P., Connolly A.M., Day J.W., Flanigan K.M., Goemans N. (2014). Ataluren treatment of patients with nonsense mutation dystrophinopathy. Muscle Nerve.

[24] Aartsma-Rus A., Ferlini A., Vroom E. (2014). Biomarkers and surrogate endpoints in duchenne: Meeting report. Neuromuscul. Disord..

[25] Ayoglu B., Chaouch A., Lochmuller H., Politano L., Bertini E., Spitali P., Hiller M., Niks E.H., Gualandi F., Ponten F. (2014). Affinity proteomics within rare diseases: A bio-nmd study for blood biomarkers of muscular dystrophies. EMBO Mol. Med..

[26] Hashim R., Shaheen S., Ahmad S., Sattar A., Khan F.A. (2011). Comparison of serum creatine kinase estimation with short tandem repeats based linkage analysis in carriers and affected children of duchenne muscular dystrophy. J. Ayub Med. Coll. Abbottabad.

[27] Rouillon J., Zocevic A., Leger T., Garcia C., Camadro J.M., Udd B., Wong B., Servais L., Voit T., Svinartchouk F. (2014). Proteomics profiling of urine reveals specific titin fragments as biomarkers of duchenne muscular dystrophy. Neuromuscul. Disord..

[28] McPherron A.C., Lawler A.M., Lee S.J. (1997). Regulation of skeletal muscle mass in mice by a new tgf-beta superfamily member. Nature.

[29] McPherron A.C., Lee S.J. (1997). Double muscling in cattle due to mutations in the myostatin gene. Proc. Natl. Acad. Sci. USA.

[30] Abe S., Soejima M., Iwanuma O., Saka H., Matsunaga S., Sakiyama K., Ide Y. (2009). Expression of myostatin and follistatin in mdx mice, an animal model for muscular dystrophy. Zool. Sci..

[31] Fukushima K., Nakamura A., Ueda H., Yuasa K., Yoshida K., Takeda S., Ikeda S. (2007). Activation and localization of matrix metalloproteinase-2 and -9 in the skeletal muscle of the muscular dystrophy dog (cxmdj). BMC Musculoskelet. Disord..

[32] Gomez D.E., Alonso D.F., Yoshiji H., Thorgeirsson U.P. (1997). Tissue inhibitors of metalloproteinases: Structure, regulation and biological functions. Eur. J. Cell Biol..

[33] Kolkenbrock H., Orgel D., Hecker-Kia A., Zimmermann J., Ulbrich N. (1995). Generation and activity of the ternary gelatinase b/timp-1/lmw-stromelysin-1 complex. Biol. Chem. Hoppe Seyler.

[34] Borden P., Heller R.A. (1997). Transcriptional control of matrix metalloproteinases and the tissue inhibitors of matrix metalloproteinases. Crit. Rev. Eukaryot. Gene Expr..

[35] Cynthia Martin F., Hiller M., Spitali P., Oonk S., Dalebout H., Palmblad M., Chaouch A., Guglieri M., Straub V., Lochmuller H. (2014). Fibronectin is a serum biomarker for duchenne muscular dystrophy. Proteomics Clin. Appl..

[36] Hu J., Kong M., Ye Y., Hong S., Cheng L., Jiang L. (2014). Serum mir-206 and other muscle-specific micrornas as non-invasive biomarkers for duchenne muscular dystrophy. J. Neurochem..

[37] Cacchiarelli D., Legnini I., Martone J., Cazzella V., D’Amico A., Bertini E., Bozzoni I. (2011). Mirnas as serum biomarkers for duchenne muscular dystrophy. EMBO Mol. Med..

[38] Sun G., Haginoya K., Chiba Y., Uematsu M., Hino-Fukuyo N., Tanaka S., Onuma A., Iinuma K., Tsuchiya S. (2010). Elevated plasma levels of tissue inhibitors of metalloproteinase-1 and their overexpression in muscle in human and mouse muscular dystrophy. J. Neurol. Sci..

[39] Brunherotti M.A., Sobreira C., Rodrigues-Junior A.L., de Assis M.R., Terra Filho J., Baddini Martinez J.A. (2007). Correlations of egen klassifikation and barthel index scores with pulmonary function parameters in duchenne muscular dystrophy. Heart Lung J. Crit. Care.

[40] Rodino-Klapac L.R., Janssen P.M., Shontz K.M., Canan B., Montgomery C.L., Griffin D., Heller K., Schmelzer L., Handy C., Clark K.R. (2013). Micro-dystrophin and follistatin co-delivery restores muscle function in aged dmd model. Hum. Mol. Genet..

[41] Camerino G.M., Cannone M., Giustino A., Massari A.M., Capogrosso R.F., Cozzoli A., De Luca A. (2014). Gene expression in mdx mouse muscle in relation to age and exercise: Aberrant mechanical-metabolic coupling and implications for pre-clinical studies in duchenne muscular dystrophy. Hum. Mol. Genet..

[42] Awano H., Takeshima Y., Okizuka Y., Saiki K., Yagi M., Matsuo M. (2008). Wide ranges of serum myostatin concentrations in duchenne muscular dystrophy patients. Clin. Chim. Acta.

[43] Chien Y.H., Han D.S., Hwu W.L., Thurberg B.L., Yang W.S. (2013). Myostatin and insulin-like growth factor i: Potential therapeutic biomarkers for pompe disease. PLoS ONE.

[44] Hansen J., Brandt C., Nielsen A.R., Hojman P., Whitham M., Febbraio M.A., Pedersen B.K., Plomgaard P. (2011). Exercise induces a marked increase in plasma follistatin: Evidence that follistatin is a contraction-induced hepatokine. Endocrinology.

[45] Ma K., Mallidis C., Artaza J., Taylor W., Gonzalez-Cadavid N., Bhasin S. (2001). Characterization of 5ʹ-regulatory region of human myostatin gene: Regulation by dexamethasone *in vitro*. Am. J. Physiol. Endocrinol. Metab..

[46] Qin J., Du R., Yang Y.Q., Zhang H.Q., Li Q., Liu L., Guan H., Hou J., An X.R. (2013). Dexamethasone-induced skeletal muscle atrophy was associated with upregulation of myostatin promoter activity. Res. Vet. Sci..

[47] Miyamoto T., Carrero J.J., Qureshi A.R., Anderstam B., Heimburger O., Barany P., Lindholm B., Stenvinkel P. (2011). Circulating follistatin in patients with chronic kidney disease: Implications for muscle strength, bone mineral density, inflammation, and survival. Clin. J. Am. Soc. Nephrol..

[48] Mathews K.D., Cunniff C., Kantamneni J.R., Ciafaloni E., Miller T., Matthews D., Cwik V., Druschel C., Miller L., Meaney F.J. (2010). Muscular dystrophy surveillance tracking and research network (md starnet): Case definition in surveillance for childhood-onset duchenne/becker muscular dystrophy. J. Child. Neurol..

[49] Vazquez-Cardenas N.A., Ibarra-Hernandez F., Lopez-Hernandez L.B., Escobar-Cedillo R.E., Ruano-Calderon L.A., Gomez-Diaz B., Garcia-Calderon N., Carriedo-Davila M.F., Rojas-Hurtado L.G., Luna-Padron E. (2013). Diagnosis and treatment with steroids for patients with duchenne muscular dystrophy: Experience and recommendations for mexico. Rev. Neurol..

[50] Bushby K., Finkel R., Birnkrant D.J., Case L.E., Clemens P.R., Cripe L., Kaul A., Kinnett K., McDonald C., Pandya S. (2010). Diagnosis and management of duchenne muscular dystrophy, part 1: Diagnosis, and pharmacological and psychosocial management. Lancet. Neurol..

[51] Gustincich S., Manfioletti G., Del Sal G., Schneider C., Carninci P. (1991). A fast method for high-quality genomic DNA extraction from whole human blood. Biotechniques.

[52] Lopez-Hernandez L.B., Gomez-Diaz B., Luna-Angulo A.B., Anaya-Segura M., Bunyan D.J., Zuniga-Guzman C., Escobar-Cedillo R.E., Roque-Ramirez B., Ruano-Calderon L.A., Rangel-Villalobos H. (2015). Comparison of mutation profiles in the duchenne muscular dystrophy gene among populations: Implications for potential molecular therapies. Int. J. Mol. Sci..

[53] Gomez-Diaz B., Rosas-Vargas H., Roque-Ramirez B., Meza-Espinoza P., Ruano-Calderon L.A., Fernandez-Valverde F., Escalante-Bautista D., Escobar-Cedillo R.E., Sanchez-Chapul L., Vargas-Canas S. (2012). Immunodetection analysis of muscular dystrophies in mexico. Muscle Nerve.

[54] Janssen B., Hartmann C., Scholz V., Jauch A., Zschocke J. (2005). Mlpa analysis for the detection of deletions, duplications and complex rearrangements in the dystrophin gene: Potential and pitfalls. Neurogenetics.

