# Supplementary material for: Non-Invasive Biomarkers for Duchenne Muscular Dystrophy and Carrier Detection
Source: Molecules. 2015 Jun 17;20(6):11154–72. doi: 10.3390/molecules200611154 (PMC6272409; doi:10.3390/molecules200611154)
Supplement: Supplementary file 1 [file molecules-20-11154-s001.pdf]

Supplementary Materials

Table S1. Clinical parameters of DMD steroid naïve ambulant patients.

Patient	Mutation	Age (Years)	NSAA	6 MW	Barthel	Brook U	Brook L	Vignos	T10mw	T10mr	Gowers	Stair	Chair	Shirt
**P1**	c.6439-?_7660+?del	3.1	18	-	-	1	3	2	-	-	-	-	-	-
**P2**	dystrophin IMF	5.6	27	-	-	1	2	2	-	0.72	4.75	4.96	1.35	18
**P3**	c.7661-?_8217+?del	6.2	18	330	85	1	2	3	0.8	1.07	11	56	3	16
**P4**	c.6439-?_6614+?del	6.5	10	415	95	1	2	2	0.6	1.3	10	5	3	11
**P5**	c.6615-?_7200+?del	6.9	-	-	90	1	3	3	0.6	1.3	26	14	1	17
**P6**	dystrophin IMF	6.9	13	286	80	1	3	2	-	0.73	NA	9.7	2.7	11.1
**P7**	c.265-?_530+?del	7	-	-	-	-	-	-	-	-	-	-	-	-
**P8**	c.6913-?_7660+?del	7.4	0	-	55	1	3	0.4	0.6	NA	44	10	35	0
**P9**	c.5326-?_5448+?dup	7.5	14	342	90	1	3	3	0.9	1.3	7	5	2	13
**P10**	c.6615-?_7660+?del	7.8	17	288	-	1	2	2	-	0.8	7	6	1	9
**P11**	c.6615-?_7872+?del	7.8	20	275	-	1	2	2	-	1.1	15	-	2	7
**P12**	c.32-?_93+?dup	7.9	13	320	95	1	3	3	0.8	1.5	13	6	3	20
**P13**	c.32-?_93+?dup	8	-	224	70	1	3	3	0.6	0.7	NA	27	3	27
**P14**	c.6439-?_6614+?del	8.2	12	279	95	1	3	3	0.9	1.5	9	271	2	19
**P15**	c.6439-?_7660+?del	8.3	11	343	90	1	3	3	0.8	1.1	32	15	4	18
**P16**	c.6439-?_8027+?del	8.3	-	-	-	3	3	-	-	-	14	-	1	-
**P17**	dystrophin IMF	8.8	13	286	-	1	1	2	-	0.73	NA	9.7	2.7	11.1
**P18**	c.7099-?_7309+?del	10.2	-	-	-	9	3	7	-	0.6	NA	NA	-	-
**P19**	dystrophin IMF	7.0	-	-	-	-	-	-	-	-	-	-	-	-
**P20**	c.6439-?_8390+?del	10.5	17	288	-	1	2	2	-	0.8	7	6	1	9
**P21**	c.5923-?_6290+?del	10.6	-	-	90	1	3	3	0.7	1.07	6	6	2	11
**P22**	dystrophin IMF	10.7	8	114	85	1	3	3	0.7	0.9	NA	15	4	10
**P23**[¥]	dystrophin IMF	10.7	27	130	90	1	2	2	NA	0.1	13	NA	2	7
**P24**	c.6439-?_7660+?del	11.7	-	-	50	2	5	5	0.2	0.2	84	NA	NA	25
**P25**	dystrophin IMF	12	-	-	-	1	2	2	-	-	6	-	-	-

[¥]P23 was taken into account only in correlation between MMP-9 serum levels, clinical parameters and age.

Table S2. Comparison of protein serum levels in LGMD groups matched by gender and age, respectively.

Protein	LGMD (Male)			LGMD (Female)		
	Patients *n* = 3 Mean (±SD)	Controls *n* = 4 Mean (±SD)	*P* Value	Patients *n* = 4 Mean (±SD)	Controls *n* = 17 Mean (±SD)	*P* value
**MMP-9**	517.00 (318.5)	165.3 (114.18)	0.090	707.05 (333.07)	705.76 (550.15)	0.994
**MMP-2**	241.55 (68.51)	519.83 (112.33)	0.013 *	ND	ND	ND
**FSTN**	1.686 (0.528)	0.715 (0.1521)	0.016 *	1.577 (0. 974)	¶ 1.830 (0.491)	0.519

* denotes statistical significance, ¶ *n* = 9, ND = Non determined.

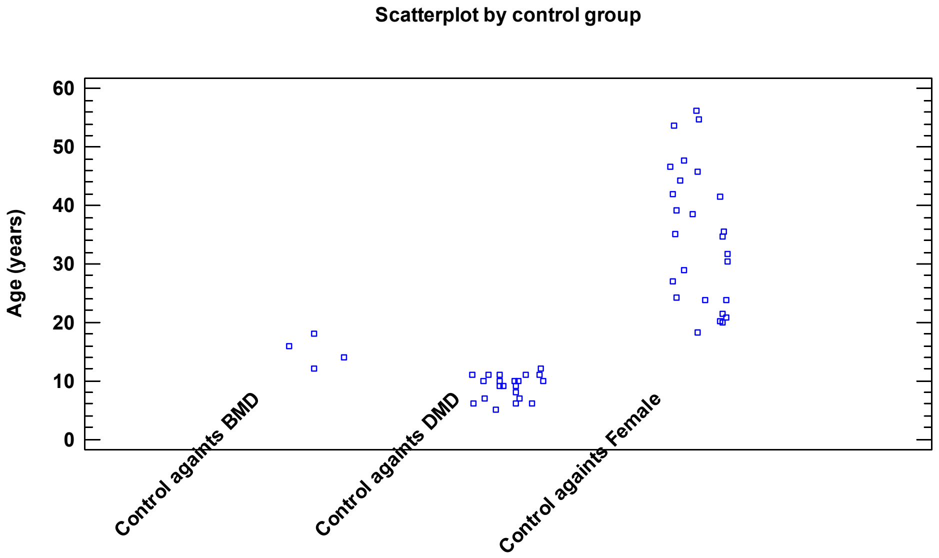

Figure S1. Dot Plot of age by controls used against DMD, BMD and carriers.
